# Costs and usage of healthcare services before and after open bariatric surgery

**DOI:** 10.1590/S1516-31802011000500003

**Published:** 2011-09-01

**Authors:** Silvana Marcia Bruschi Kelles, Sandhi Maria Barreto, Henrique Leonardo Guerra

**Affiliations:** I MSc. Health Technology Assessment Group Coordinator in Unimed, Belo Horizonte, and Researcher at Hospital das Clínicas, Universidade Federal de Minas Gerais (UFMG), Belo Horizonte, Minas Gerais, Brazil.; II PhD. Associate Professor of Epidemiology, Universidade Federal de Minas Gerais (UFMG), and Coordinator of the Longitudinal Adult Health Study (ELSA Brazil), UFMG, Belo Horizonte, Minas Gerais, Brazil.; III PhD. Assistant Professor at Pontifícia Universidade Católica de Minas Gerais. Director of Epicentro, Minas Gerais, Brazil.

**Keywords:** Obesity, morbid, Gastric bypass, Health care costs, Morbidity, Bariatric surgery, Obesidade mórbida, Derivação gástrica, Custos de cuidados de saúde, Morbidade, Cirurgia bariátrica

## Abstract

**CONTEXT AND OBJECTIVE:**

Morbidly obese individuals are major consumers of healthcare services, with high associated costs. Bariatric surgery is an alternative for improving these individuals’ comorbidities. There are no studies comparing costs before and after bariatric surgery in Brazil. The aim here was to analyze results relating to healthcare usage and direct costs among morbidly obese patients undergoing bariatric surgery.

**DESIGN AND SETTING:**

Historical cohort study on patients receiving healthcare through a private health plan in Belo Horizonte, Minas Gerais.

**METHODS:**

All healthcare services and their associated costs were included in the analysis: hospitalization, hospital stay, elective outpatient consultations, emergency service usage and examinations. The analyses were treated as total when including the whole years before and after surgery, or partial when excluding the three-month periods adjacent to the operation.

**RESULTS:**

For 382 obese patients who underwent open bariatric operations, there were 53 hospitalizations one year before and 95 one year after surgery (P = 0.013). Gastrointestinal complications were the main indications for post-procedure hospitalizations. The partial average cost almost doubled after the operation (US$ 391.96 versus US$ 678.31). In subgroup analysis, the costs from patients with gastrointestinal complications were almost four times greater after bariatric surgery. Even in the subgroup without complications, the partial average cost remained significantly higher.

**CONCLUSION:**

Although bariatric surgery is the only path towards sustained weight loss for morbidly obese patients, the direct costs over the first year after the procedure are greater. Further studies, with longer follow-up, might elucidate whether long-term reversal of this trend would occur.

## RESUMO

**CONTEXTO E OBJETIVO:**

Indivíduos obesos mórbidos são grandes consumidores de serviços de saúde, com altos custos associados. A cirurgia bariátrica é uma alternativa para melhorar suas comorbidades. Não existem estudos comparando custos antes e após a cirurgia bariátrica no Brasil. O objetivo deste estudo foi analisar os resultados assistenciais e gastos diretos de pacientes obesos mórbidos submetidos à cirurgia bariátrica.

**TIPO DE ESTUDO E LOCAL:**

Coorte histórica de pacientes assistidos por um plano de saúde na cidade de Belo Horizonte, Minas Gerais.

**MÉTODOS:**

Foram incluídos na análise todos os serviços de saúde e seus custos associados: hospitalizações, permanência hospitalar, consultas eletivas e de emergência e exames. A análise foi tratada como total quando incluía todo o ano anterior e posterior à cirurgia, e parcial quando excluía os trimestres adjacentes à cirurgia.

**RESULTADOS:**

Para 382 pacientes obesos operados, houve 53 internações um ano antes e 95 um ano após a cirurgia (P = 0,013). As principais indicações para internação pós-procedimento foram complicações relacionadas ao trato gastrointestinal. O custo parcial médio quase dobrou no período pós-operatório (US$ 391,96 *versus* US$ 678,31). Em análise de subgrupo, pacientes com complicações do trato gastrointestinal apresentaram custos quase quatro vezes maiores no período pós-bariátrica, e mesmo no subgrupo sem estas complicações, o custo parcial médio permaneceu significativamente maior.

**CONCLUSÃO:**

Embora a cirurgia bariátrica seja o único caminho para perda de peso sustentada nos pacientes obesos mórbidos, os custos diretos até um ano pós-procedimento são maiores. Novos estudos, com maior tempo de acompanhamento, poderão verificar se essa tendência se reverte no longo prazo.

## INTRODUCTION

Obesity has assumed alarming proportions throughout the world over the last decade. Many factors have contributed towards this growth: not only the increasing consumption of hypercaloric food and reduction in physical activity, but also genetic causes.^1^ Obesity is associated with five of the most frequent causes of death among adults worldwide. Data from the Framingham study show that a 10% reduction in body weight corresponds to a 20% reduction in the risk of developing coronary disease.^1^ Moreover, decreased occurrences of comorbidities such as arterial hypertension, diabetes mellitus, sleep apnea, osteoarthritis and other degenerative conditions are seen, along with weight loss.

In the United States, obesity (body mass index, BMI ≥ 30 kg/m²) affects 31% of the population, of which 5% are morbidly obese (BMI ≥ 40 kg/m²).^2^ Obesity is the second biggest preventable cause of death in the United States.^3^ In Brazil, the prevalence of obesity among adults is 13% and 9% for females and males, respectively, with 0.64% presenting morbid obesity, i.e. approximately 609,000 people. This prevalence has increased by 100% in about a decade.^4^ The growth of morbid obesity is one of the major challenges to public health.

Life expectancy among morbidly obese individuals may be reduced by 5 to 20 years.^5^ Mortality among such individuals may be 12 times higher in the third or fourth decade of their lives.^6^

Prevention is certainly the most effective method for approaching the problem,^7^ but currently there is a huge contingent of people who are already morbidly obese and require therapeutic intervention. Worldwide experience has shown that there are serious limitations to the results from conservative treatment of morbid obesity, with success rates of only around 10%.^2^ Thus, bariatric surgery has assumed a leading role in managing morbidly obese individuals, with several surgical techniques available.

Scientific evidence has shown that bariatric surgery leads to sustained loss of excess weight, of around 20 kg to 30 kg over eight years of follow-up.^8,9^ Data from the Swedish Obesity Study (SOS)^8,9^ showed a dramatic reduction in the risk of diabetes type 2 among the operated group, but the reduction in hypertension was not sustained over eight years of follow-up. The benefits regarding hyperuricemia, some types of dyslipidemia and sleep apnea and the improvement in dyspnea symptoms and chest pain are still evident after ten years of follow-up.

Consistent loss of excess weight after surgery has been well established in the literature. It continues for up to two years, by which time patients have lost 89% of their excessive weight.^10^ After that, there is a weight stability phase, which can last for over 11 years,^9^ followed by a slight weight regain. Even with a gradual rise in body weight, patients usually do not reach the preoperative weight. Such weight regains confirm that obesity is a chronic and progressive disease that is very resistant to conservative treatment and even to surgery.^11^

The costs involved in managing morbidly obese patients are a challenge both for public and for private medical care. The expenditure is high, since this is a chronic and highly prevalent disease that is difficult to manage and often associated with clinically relevant comorbidities. In 2000, it was estimated that obesity had contributed towards 400,000 deaths in the United States.^12^ The healthcare expenditure on diseases relating to morbid obesity or associated with their medical conditions among Americans was estimated to be 100 billion dollars during the year 1995.^13^

In Brazil, bariatric surgery is covered by the National Health System (Sistema Único de Saúde, SUS). There is one reference center for bariatric surgery for every four million inhabitants. With such limited provision, there are long waiting lists for candidates, and surgery tends to be performed only when patients have already reached more advanced stages of morbid obesity and associated comorbidities.

In addition to public healthcare services, about 22% of the Brazilian population (40 million people) has private healthcare insurance. Patients covered by private health plans have rapid access to bariatric surgery if they meet the clinical inclusion criteria.

There is a lack of studies on the direct costs of bariatric surgery worldwide, and the present authors did not find any study involving analysis on Brazilian patients and costs.

## OBJECTIVE

The objective of the present study was to assess the healthcare usage profiles and related costs among patients before and after open bariatric surgery. We also investigated whether costs and healthcare usage were associated with individual characteristics such as age, gender, BMI and presence of comorbidities.

## METHODS

This was a historical cohort of patients who underwent surgery in order to treat morbid obesity between January and December 2005 and who were covered by one of the largest Brazilian health maintenance organizations (HMOs). All patients who had been covered by the HMO for at least one year before and one year after the surgery or who had died within one year after surgery were eligible to participate in the study. Records from a medical audit conducted by the HMO staff before bariatric surgery containing data on age, sex, BMI, results from preoperative tests and comorbidities, were available for each patient. Information on healthcare service usage and costs were available from the HMO database.

All the patients underwent conventional bariatric surgery (not laparoscopic) using the Roux-en-Y gastric bypass technique at hospitals in the Brazilian city of Belo Horizonte. The cost was defined as the amount spent by the HMO on paying for procedures, therapies, fees and examinations. Amounts were presented in United States dollars (US$) using the average exchange rate from 18 months before June 2005 for the preoperative period (2.83 reais = 1 US$) and 18 months after June 2005 for the postoperative period (2.24 reais = 1 US$).^14^ For the cost of the bariatric surgery itself, the exchange rate was the average for June 2005 (2.37 reais = 1 US$).^15^

The costs of the health services used were calculated based on the HMO data for the year before and the year after surgery. The total cost was the sum of all expenditures throughout the year before and the year after the surgery and the partial cost was equal to the total cost minus the cost associated with expenditures over the three-month periods immediately before or after the surgery. These three-month periods adjacent to the surgery were considered to be directly related to the preparation for and the immediate consequences of bariatric surgery. We tried to obtain the patient's pattern of usage with minimal interference from the surgery itself.

Pre and postoperative hospital stays were categorized according to number, length of hospital stay and indications for hospitalization, as described in the 10^th^ revision of the International Classification of Diseases (ICD-10) or according to the procedure requested. Reasons for hospitalization were grouped as follows: a) procedures for the gastrointestinal tract or complications relating to bariatric surgery; b) elective surgical procedures (excluding plastic surgery); c) orthopedic procedures; d) clinical admission unrelated to bariatric surgery; e) cardiovascular procedures; and f) other reasons. Hospital stays due to pregnancy and childbirth was excluded because of their volitional nature.

Furthermore, data relating to outpatient visits, emergency consultations, imaging examinations (radiography, ultrasound, computed tomography, CT, or magnetic resonance) and laboratory tests (pathological and clinical pathological analyses) were also brought together and presented.

This work was submitted to and approved by the Research Ethics Committee of Universidade Federal de Minas Gerais (UFMG) (COEP 412/2007). Since this study consisted of secondary data analysis, the requirement for patient consent was waived.

Central trend measurements were estimated for continuous variables, with calculation of means, medians and standard deviations (SD). Two-tailed paired t tests were used to compare the mean frequencies of admissions, consultations, examinations and other events between females and males, in the year before and the year after surgery. BMI was categorized as < 50 kg/m² or ≥ 50 kg/m², and age as below or above 50 years. Hospital stay, costs, number of examinations and consultations were categorized as above or below the 90^th^ percentile. For categorical variables, we used the chi-square or Fisher's exact test. Associations between the response-variables of “use of services above the 90^th^ percentile”, “previous hospitalization” and “after surgery” and the independent variables of age, sex, BMI and comorbidities were investigated by using multiple logistic regression. All variables with significance level lower than 0.20 were entered into the final model, but only the variables that continued to be associated at significance levels below 0.05 were kept in it. Data were entered into Epi Info 6.04 and analyzed using the Stata statistics and data analysis package, version 9.2.

## RESULTS

In 2005, 535 bariatric surgery procedures were performed with coverage from the HMO in Belo Horizonte. Out of that total, 153 (20%), did not fulfill the criteria for inclusion in the study: 112 (73.2%) had joined the HMO less than one year before the surgery and 41 (26.8%) had left it less than one year after surgery. The reasons why these 41 patients left the HMO were administrative, for example: the company at which they were employed canceled the contract with the HMO; financial problems; job loss or moving to another city.

Thus, 382 patients fulfilled the inclusion criteria for the study. Table 1 shows that, in the preoperative phase, men had significantly greater BMI than women.

**Table 1.** Patients’ characteristics according to gender

Characteristic	Female n = 317 (83%)	Male n = 65 (17%)	General n = 382 (100%)	P value
Age in years: mean (SD) Median	38.2 (10.7) 36.9	36.5 (10.2) 35.9	38.0 (10.6) 36.2	0.24
BMI (kg/m²): mean (SD) Median	42.5 (4.5) 41.0	45.0 (5.3) 44.0	43.0 (4.7) 42.0	0.00
Hospital stay* (days): mean (SD) Median	4.3 (13.9) 3.1	3.6 (2.0) 3.2	4.2 (12.7) 3.1	0.67
Presence of at least one comorbidity: n (%)	175/314 (55.7)	39/65 (60.0)	214/379 (56.5)	0.29
Arterial hypertension: n (%)	112/314 (35.7)	31/65 (47.7)	143/379 (37.7)	0.07
Diabetes mellitus: n (%)	38/314 (12.1)	10/65 (15.4)	48/379 (12.7)	0.47
Sleep apnea: n (%)	8/314 (2.5)	3/65 (4.6)	11/379 (2.9)	0.41†
Arthropathy: n (%)	60/314 (19.1)	7/65 (10.8)	67/379 (17.7)	0.11
Others: n (%)	3/314 (1.0)	2/65 (3.0)	5/379 (1.3)	0.20†
Deaths within 30 days: n (%)	1/317 (0.3)	1/65 (1.5)	2/382 (0.5)	0.31†
Deaths within one year: n (%)	2/317 (0.6)	2/65 (3.1)	4/382 (1.0)	0.14†

* Hospital stay for bariatric surgery;

† Fisher's exact test;

SD = standard deviation; BMI = body mass index.

The 30-day mortality rate post-surgery was 0.5%, with two deaths, one from anastomotic leak and sepsis and one from pulmonary thromboembolism. The cumulative mortality up to one year was 1%, with two more deaths, one after 54 days and another after six months. Both of these patients remained hospitalized from the time of the surgery until their deaths, due to complications subsequent to anastomotic leak.

**Figure 1.**
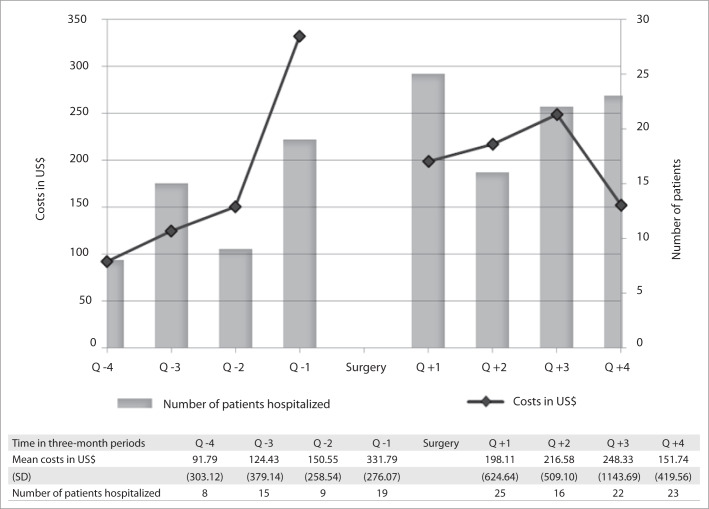
Number of hospitalized patients and mean cost relating to patients who underwent bariatric surgery, according to three-month periods (quarter-year, Q), one year before and one year after surgery. Q −1 refers to the first three-month period immediately before surgery; Q −2 refers to the second trimester before the surgery and so on. Q +1, +2 and so on refer to the successive three-month periods after the surgery). US$ = United States dollars; SD = standard deviation.

The mean cost of bariatric surgery was US$ 3,227.16 and the mean total cost of healthcare services per patient, in two years, including surgery, was US$ 4,880.34 (SD = US$ 3,116.10). The costs in each three-month period comprised the sum of all costs covered by the HMO in relation to hospital stay, emergency services, outpatient visits, laboratory tests and imaging examinations. Figure 1 shows the number of hospitalized patients and the mean costs of patients, per three-month period, in the year before and the year after the bariatric surgery. Excluding the two three-month periods adjacent to the surgery, hospitalization nearly doubled between the two periods compared: 32 preoperative versus 61 postoperative hospital admissions.

The mean hospital stay for bariatric surgery was 4.2 days (SD = 12.7 days), ranging from 1.7 to 240 days, and the median was 3.1 days.

During the year before bariatric surgery there were 53 hospitalizations, involving 51 patients (13.4%) and 255 days in hospital. The presence of diabetes and arthropathy showed statistically significant associations with these hospitalizations in multiple logistic regression analysis.

During the year after surgery, there were 95 hospitalizations involving 86 patients (22.5%) and 568 days in hospital. Age ≥ 50 years was the only statistically significant variable associated with hospitalization after surgery.

The difference between the numbers of hospitalizations before and after surgery was statistically significant (P = 0.013), with a chance of hospitalization post-surgery that was 2.7 times greater (95% confidence interval, CI: 1.18-4.74).

Table 2 shows the numbers of hospitalizations, categorized by indication, one year before and one year after bariatric surgery. Some hospitalizations were due to surgical complications, while others were due to preexisting conditions that could be better managed after weight loss, such as treatment for urinary incontinence, varicose veins in the lower limbs and knee arthropathy.

**Table 2.** Numbers and indications for hospitalization, one year before and one year after bariatric surgery

Admission causes one year before surgery	Number of hospitalizations	Admission causes one year after surgery	Number of hospitalizations
**Patients with gastrointestinal tract procedures or complications relating to bariatric surgery procedure**			
Laparoscopic surgery	6	Laparoscopic surgery	13
		Laparotomy surgery	12
Endoscopic interventions	2	Endoscopic interventions	2
		Hospitalizations due to surgical complications	30
**Patients with elective surgical procedures plastic surgery excluded)**			
Breast intervention	1	Breast intervention	1
Genitourinary tract intervention	8	Genitourinary tract intervention	14
**Patients with orthopedic surgical procedures**			
Fractures	1	Fractures	1
Limb arthropathy	4	Limb arthropathy	4
Spinal arthropathy	7	Spinal arthropathy	4
**Patients with clinical hospital admissions**			
Infectious diseases	3	Infectious diseases	2
Chronic degenerative diseases	3	Chronic degenerative diseases	4
Psychiatric hospitalizations	5	Psychiatric hospitalizations	2
**Patients with cardiovascular procedures**			
Cardiac procedures	2	Cardiac procedures	1
Lower-limb procedures	6	Lower-limb procedures	3
**Patients with other reasons for hospitalization**			
Tumors (benign)	3	Tumors (benign)	1
Trauma	1	Trauma	1
Pulmonary thromboembolism	1		
Hospitalization total	53		95

The most frequent reasons for hospitalization after bariatric surgery were: clinical partially obstructed bowel (16%), cholecystectomy (16%), venous thrombosis or pulmonary thromboembolism (16%), dehydration and vomiting (14%) and surgical acute abdomen (14%).

The number of consultations fell significantly in the postoperative year: from 4,639 to 3,632 visits (P < 0.001). But when the three-month periods adjacent to the surgery were excluded, it was seen that there was a significant increase in the mean number of postoperative outpatient visits (Table 3).

**Table 3.** Changes in healthcare service usage and costs from before to after the operation

Variables	Before operation	After operation	Comparison between means of paired samples		
	Mean number	Mean number	Difference between means (before/after)	95% CI	P value
**Elective consultation**					
Whole year	12.1	9.5	2.64	2.16 to 3.11	< 0.001
Partial year	6.1	6.9	−0.82	−1.27 to 0.38	< 0.001
**Emergency consultation**					
Whole year	1.3	1.2	0.05	−0.15 to 0.26	0.594
Partial year	0.9	0.9	> 0.01	−0.16 to 0.17	0.975
**Laboratory tests**					
Whole year	33.5	31.0	2.46	0.10 to 5.03	0.059
Partial year	13.9	24.2	−10.3	−12.4 to −8.17	< 0.001
**Imaging examinations**					
Whole year	3.7	2.0	1.76	1.47 to 2.06	< 0.001
Partial year	1.8	1.5	0.24	−0.001 to 0.49	0.051
**Costs (US$) for all 382 patients**	**Mean cost**	**Mean cost**	**Difference between mean costs**	**95% CI**	**P value**
Whole year	756.94	896.23	−139.29	−375.19 to 96.61	0.246
Partial year	391.96	678.31	−286.35	−473.28 to −99.42	0.003
**Costs (US$) for 43 patients who underwent gastrointestinal tract procedure or had complications related to bariatric surgery procedure**					
Whole year	784.01	2,897.20	−2,113.18	−3,767.58 to −458.79	0.014
Partial year	460.03	1,979.59	−1,519.56	−2,824.27 to −214.85	0.024
**Costs (US$) for the other 339 patients without intervention or with other interventions**					
Whole year	687.80	554.82	132.97	27.75 to 238.20	0.013
Partial year	355.00	448.00	−92.98	−185.86 to −0.09	0.05

CI = confidence interval; US$ = United States dollars.

Whole year: measurement considering four three-month periods both before and after surgery; Partial year: measurement excluding the three-month periods immediately before and after the surgery.

With regard to elective consultations with HMO doctors during the preoperative period, excluding the three-month period immediately before the procedure, 28 (7.3%) patients did not have any elective consultation, 208 (54.4%) had up to six and 146 (38.6%) had seven or more. The factors statistically associated with the 90^th^ percentile (13 consultations) were female sex, diabetes, arthropathy and age ≥ 50 years in multivariate logistic regression analysis. Patients presenting these characteristics had 12 times more chances of being a very high outpatient care user.

During the postoperative period, excluding the three-month period immediately after the procedure, seven (1.8%) patients had no consultation, 207 (54.2%) had up to six and 168 (44.0%) had seven or more consultations. The 90^th^ percentile (13 visits) was statistically associated with hypertension and diabetes, and hypertension was the only variable that remained significant in the multivariate analysis.

The number of visits to emergency services before and after surgery did not differ significantly, either considering the whole year or excluding the three-month periods adjacent to the surgery.

Considering the entire year, the mean numbers of pre and postoperative laboratory tests were not statistically different. After exclusion of the three-month periods adjacent to the surgery, there was a significant increase in the number of laboratory tests during the postoperative period (P ≤ 0.001) (Table 3). The mean number of imaging examinations decreased in the postoperative period, considering both the whole year and the year without the three-month periods adjacent to the surgery (Table 3).

A statistically significant difference was found from comparing the mean costs in the pre and postoperative periods (US$ 756.24 versus US$ 896.23, respectively) (Table 3). After exclusion of the three-month periods adjacent to the surgery, the mean cost in the preoperative period was US$ 391.96, i.e. half of what was observed in the postoperative period: US$ 678.31 (P = 0.003).

In a subgroup analysis, there were 43 patients hospitalized due to surgical complications or intervention in the gastrointestinal tract. The mean costs over the whole year before bariatric surgery were US$ 784.01 and US$ 2,897.20 (P = 0.014) and the costs excluding the three-month periods adjacent to the procedure before and after the procedure were US$ 460.03 and US$ 1,979.59, respectively (P = 0.024). The difference in mean costs between the partial time periods remained significant even for the subgroup with no complications due to bariatric surgery (Table 3).

Hypertensive patients had significantly higher costs than non-hypertensive patients. The same was found in relation to patients with diabetes, BMI ≥ 50 kg/m² and age over 50 years.

## DISCUSSION

In Brazil, to the best of our knowledge, this was the first study to assess and compare the direct costs of healthcare before and after open bariatric surgery. Our sample included individuals covered by private health insurance with easy and non-differential access to any necessary treatment or examination. The analyses showed that the patients’ consumption of resources increased in the year after surgery, despite their overall expected improvement in health profile. Hospitalizations doubled, mainly due to interventions in the gastrointestinal tract and to surgical complications. Consultations and examinations were also more frequent during postoperative period.

Mortality analysis was not a specific objective of our study, but the rates and the death causes found were consistent with those described in the literature.^8,9,16-18^

The mean amount paid for open bariatric surgery was US$ 3,227.16, representing 66% of the total healthcare cost over the two years analyzed. Over the same period, SUS paid, on average, US$ 1,380.74 for the same open surgery, i.e. less than half of what was paid by the private sector.^19^ Livingston et al.^20^ calculated the median total cost of open gastric bypass in the United States as US$ 11,157.00. Many reasons explain cost variations between countries, but they were not the focus of the present work.

Considering the whole year, the direct costs from patients who underwent bariatric surgery increased in the postoperative period and became significantly higher (P = 0.003), after removing the costs in the three-month periods adjacent to the surgery. The same occurred in the three-year follow-up cohort of Zingmond et al.^21^ and the studies by Encinosa et al.^22^ Our findings confirm that, at least over the short term, from the payer's point of view, the costs do not diminish after surgery; instead, they tend to increase. As expected, the costs in the subgroup with any type of complication after bariatric surgery or a gastrointestinal tract procedure were much higher than among those with uncomplicated procedures. Considering the total costs, the expenditure after surgery decreased among the patients without complications. However, when the costs due to healthcare usage directly relating to the surgery (three-month periods immediately before and after the procedure) were removed from the analysis, the partial expenditure after surgery was significantly greater than before it.

Christou et al.^17^ compared the expenditure on patients who underwent bariatric surgery with the expenditure on morbidly obese patients who did not undergo this procedure, and found that the direct costs relating to those who underwent the surgery went down by 45% over a five-year follow-up. Obese patients, even without surgery, are high consumers of healthcare services. In this study, only the costs up to one year after surgery were included, and therefore the long-term costs were not calculated.

Exclusion from the analysis of the healthcare costs during the trimesters adjacent to the surgery was the strategy adopted to assess the real trend in healthcare consumption. In some cases (Table 3), the direction of the variation was reversed, with regard to the numbers of elective consultations laboratory tests and imaging examinations, probably because of pre-surgery examinations.

Zingmond et al.^21^ excluded bariatric surgery from the cost calculation, but the influence of the surgery is not limited only to the period of hospitalization for the procedure itself, as shown in the present study. Hence, only through excluding both the procedures and the costs directly driven by surgery is it possible to obtain a picture of the usual healthcare consumption of morbidly obese patients.

The number of hospitalizations in the year after the surgery, both considering the entire year and excluding the three-month periods immediately before and after surgery, was higher than in the preoperative year. The number of patients hospitalized after surgery nearly doubled, in comparison with the preoperative period: 61 versus 32 patients, respectively. In the study by Christou et al.^17^ the number of post-bariatric hospitalizations decreased, but Zingmond et al.^21^ found more postoperative hospitalizations. However, in Zingmond's sample, reparative plastic surgery within one year after surgery was included in the analysis. In our study, other than the clinical and surgical hospitalizations described earlier, the HMO did not cover plastic surgery until one year after bariatric surgery, and therefore such costs were not included in our analysis.

Among the individuals who underwent the surgery, 86 (22.5%) had at least one hospitalization within the entire year after surgery. Several studies have reported readmission rates within the first year of between 10 and 19.3%.^9,21^ The most common readmission causes were procedures on the gastrointestinal tract and surgical complications. An increased risk of gastrointestinal tract problems is expected. Sjöström et al.^9^ reported that the perioperative non-fatal complications included venous thromboembolism, anastomotic leakage of wound infection, bleeding, incidental splenectomy, internal and incisional hernia and intestinal obstruction. Similar indications for readmission were found in the present study. The causes of minor complications such as nausea, vomiting or anemia, with no clinical significance that could lead to hospitalization, and of dumping syndrome (present in more than 60% of the cases),^8^ could not be measured in the present study. Similarly, it was not possible to assess whether these minor complications were the reason for consultations because there was no information about them (from the codes of the International Classification of Diseases, version 10; ICD-10), in relation to outpatient care.

Cholelithiasis was a common cause of readmission after bariatric surgery. Several authors have shown a correlation between fast weight loss after bariatric surgery and cholelithiasis. The incidence has ranged from 28% to 35% of hospitalizations.^23^ In the present study, we identified four cases of cholecystectomy in the preoperative year and nine in the year that succeeded Roux-en-Y gastric bypass. It was not possible to ascertain how many patients had undergone cholecystectomy before the study period. Even so, the procedure was two times more frequent postoperatively, which would be expected according to the literature.^23^

Arthropathy and diabetes were associated with preoperative hospitalization, while age over 50 years was the only variable that significantly increased the risk of hospital admission during the postoperative period. This can be explained by the fact that arthropathy and diabetes improve or even disappear after surgery, and no longer represent a cause for hospitalization in the postoperative year.^9,17,24^ Despite all the positive aspects of improvements in health quality, hospitalization after surgery was more frequent.

The lengths of hospital stays in the present study were in accordance with the data in most of the published studies.^16,25^ For hypertensive and super-obese patients, the risk of a longer stay in hospital was 12 times greater.

The consumption of other healthcare services, such as elective consultations and laboratory tests, excluding the three-month periods adjacent to the surgery, was significantly greater in the postoperative period. This increase was expected because the management of these patients included at least seven elective consultations with multidisciplinary staff over the first year after surgery.^26^ Although the mean number of elective consultations per year was 6.9, the subgroup analysis showed that 1.8% had no consultations at all and 207 (54.2%) had less than seven. These findings may be partially explained by the fact that some patients seek assistance from private doctors who do not have a relationship with the HMO. In such cases, the total cost (i.e. not just the costs from the HMO's point of view) could be much higher. However, it may also indicate lack of adequate care for some patients during the postoperative year.

In this study, we made an inference about the healthcare status of the morbidly obese individuals based on their consumption of healthcare services. However, unlike in other studies using administrative databases,^18,27^ we had information about BMI and previous comorbidities available, which allowed us to estimate the risks according to the patient's health profile before surgery.

This study has some limitations that should be highlighted. The evaluation period was short, considering the impact of an intervention as radical as bariatric surgery. Moreover, we were unable to assess minor complications that did not lead to hospitalization or at least to an emergency consultation paid by the HMO.

We believe that the exclusion of 20% of the patients, because they did not meet the inclusion criteria of this study, did not compromise the internal validity of the results, since the reasons for exclusion were not related to the surgery. There is no reason to infer that the excluded patients would have behaved differently with regard to the health service utilization that was the focus of the analysis presented in this work. If this study had been based on public healthcare patients, there would not have been any losses due to changes in health plan or job loss. However, we would most certainly have faced follow-up losses due to changes of address or inaccurate information in the database.

Unfortunately, the costs estimated in our study did not include expenditure made directly by the patients. In Brazil, patients who have private healthcare insurance usually pay by themselves for medicines taken at home to treat comorbidities and nutritional needs. With the improvement or even the remission of some comorbidities after bariatric surgery, personal expenditures will most probably decrease.

Obesity is a public health problem that has grown in developed countries and is tending to assume increasing importance in countries like Brazil. Morbidly obese patients tend to be frequent users of healthcare services, and bariatric surgery is an attractive path towards sustained weight loss and substantial improvement in health. However these patients will require a larger number of hospitalizations, consultations and examinations, and the costs tend to be higher after surgery. There is no study showing that, by reducing BMI, health costs will reduce. The present study had the aim of contributing towards such discussions. Improving health may not be accompanied by reducing costs, with regard to care for morbidly obese patients, at least over the short term.

Faced with the prospect of hundreds of thousands of morbidly obese individuals applying for bariatric surgery, it is important to reflect on the impact of these costs on the sustainability of public and private health services. Our findings reinforce the importance of preventive policies for obesity in order to maintain the best possible state of health among individuals and to minimize the expenditure on preventable diseases in the population as a whole.

## CONCLUSION

The present study shows that healthcare use and related costs up to one year after open bariatric surgery are higher than in the year before the procedure. Further studies are needed to elucidate whether this trend of increasing costs would be maintained over a longer follow-up period. The results also show that patients presenting hypertension, diabetes, BMI ≥ 50 kg/m² and aged 50 years and above had significantly higher costs than patients without these conditions.
